# Acute pancreatitis due to pancreatic hydatid cyst: a case report and review of the literature

**DOI:** 10.1186/1749-7922-7-7

**Published:** 2012-03-24

**Authors:** Amin Makni, Mohamed Jouini, Montassar Kacem, Zoubeir Ben Safta

**Affiliations:** 1 Department of Surgery 'A', La Rabta Hospital, Tunis, Tunisia

**Keywords:** Hydatid cyst, Pancreas, Pancreatectomy, Pancreatitis

## Abstract

Hydatid disease is a major health problem worldwide. Primary hydatid disease of the pancreas is very rare and acute pancreatitis secondary to hydatid cyst has rarely been reported. We report the case of a 38-year-old man who presented acute pancreatitis. A diagnosis of hydatid cyst of the pancreas, measuring 10 cm, was established by abdominal computed tomography before surgery. The treatment consisted of a distal pancreatectomy. The postoperative period was uneventful. Additionally, a review of the literature regarding case reports of acute pancreatitis due to pancreatic hydatid cyst is presented.

## Background

Hydatid disease caused by the larval stage of the Echinococcus parasite is a public health problem in endemic countries, especially in Tunisia. Hydatid disease can involve any organ. The liver is the most common organ involved and, together with the lungs, account for 90% of cases. Other involved sites (less than 10% of cases) are muscles, bones, kidneys, brain, and spleen. Pancreatic hydatid cysts are rare, accounting for less than 1% of cases [1,2]. Isolated involvement of the pancreas is unusual, and acute pancreatitis secondary caused by primary pancreatic hydatid cyst has rarely been reported (less than 2% of cases in endemic areas) [3]. To our knowledge, 8 cases have been reported in the literature [4-11]. We reviewed and summarized the findings from reported cases of hydatid acute pancreatitis as indicated in the English literature, as well as presenting the findings from our case (see Table 1). Only one article was not available [7] and was not included in Table 1.

**Table 1:** Up-to-date review of cases of hydatid acute pancreatitis

Case n°	Source	Year	Age (sex)	Location	Size (mm)	Type of the pancreatitis	Pathogenesis¥	Surgical treatment	Follow-up (months)
1	Augustin et al. [4]	1984	30 (male)	Body	...	...	Opening	Left pancreatectomy+splenectomy	...

2	Papadimitriou [5]	1987	35 (male)	Head	50	Edematous	Opening	cyst fenestration	12

4	Ozmen et al. [7]	2005	18 (female)	Head	43	Necrotizing	Compression	Total cystectomy	16

5	Pouget et al. [8]	2009	29 (male)	Body	30	Edematous	Opening	Left pancreatectomy+splenectomy	3
6	Diop et al. [9]	2010	29 (male)	Tail	80	Edematous	Opening	Left pancreatectomy	48
7	Karakas et al. [10]	2010	18 (male)	Body	70	Edematous	Opening	cyst fenestration	4
8	Chammakhi et al. [11]	2010	32 (Female)	Tail	80	Necrotizing	Opening	Left pancreatectomy+splenectomy	6
9	Present case	2011	38 (male)	Body	100	Edematous	Opening	Left pancreatectomy+splenectomy	3

**¥ Pathogenesis: **Opening of the hydatid cyst in the main pancreatic duct or compression of the main pancreatic duct by the pancreatic hydatid cyst

Missing data

## Case presentation

**Figure 1:**
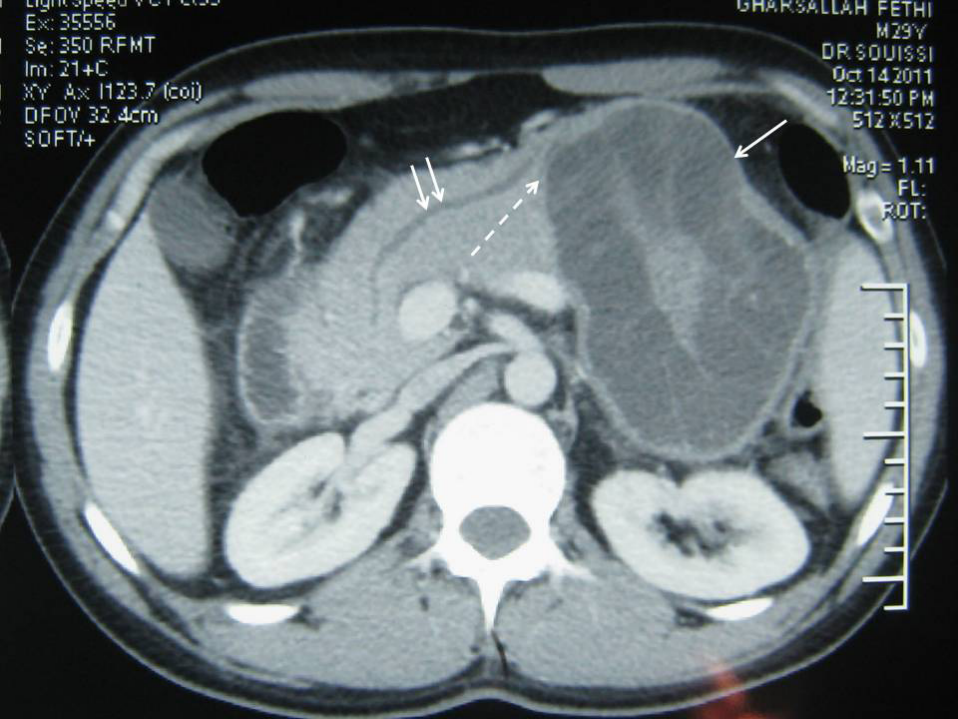
**Abdominal CT-scan shows a pancreatic cystic mass of 10 cm, with a clean and calcified wall and containing daughter cysts (one arrow)**. The main pancreatic duct is dilated (two arrows). Between the main pancreatic duct and the cyst, abdominal CT-scan shows a detachement of the hydatid membrane in the pancreatic cyst (dotted arrow).

**Figure 2:**
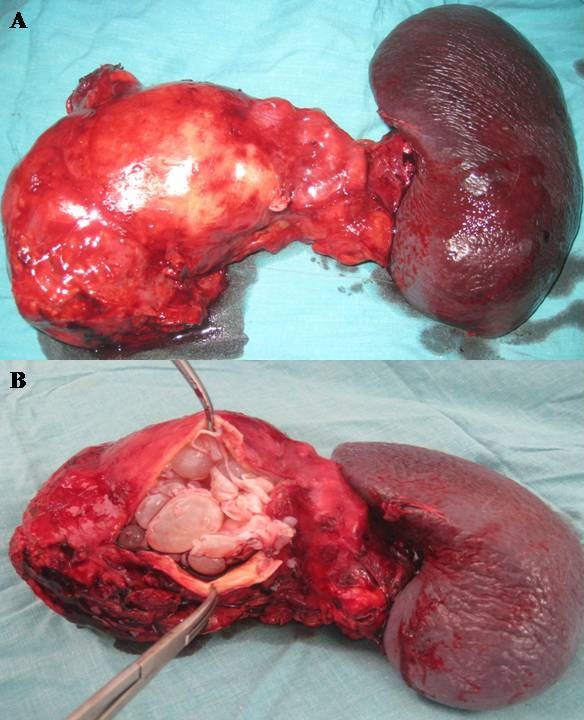
**Specimen's photograph**. **A- **A specimen of the left pancreatectomy with splenectomy, with a tumor in the corpus of the pancreas. **B- **At the opening of the cyst, we see its own wall and daughter cysts.

**Figure 3:**
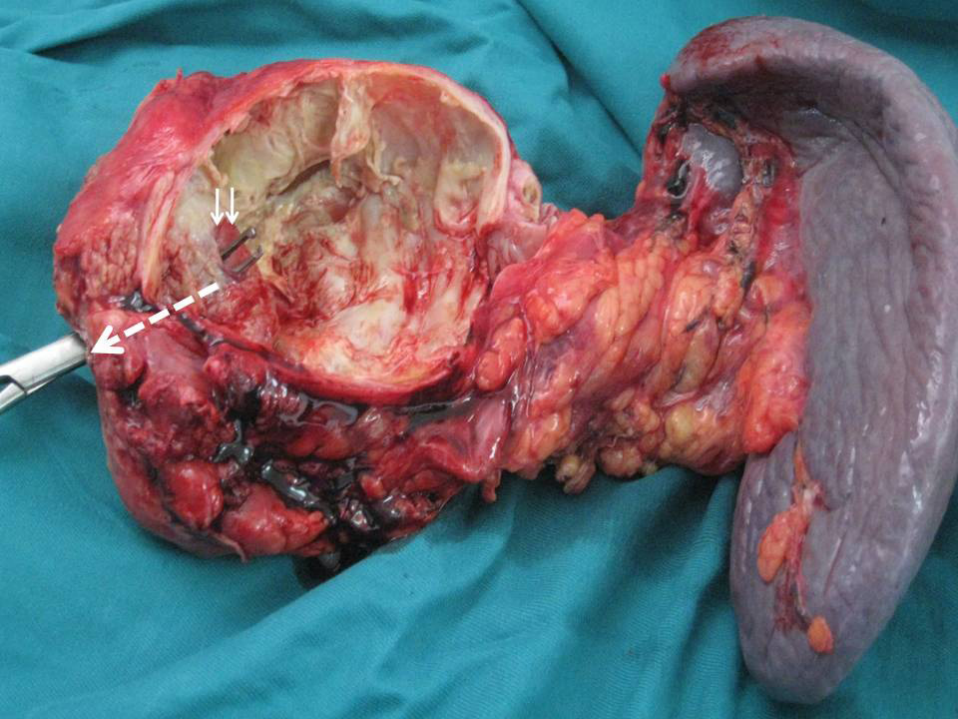
**Specimen's photograph shows a fistula between the pancreatic hydatid cyst and the main pancreatic duct (two arrows)**. The dotted arrow indicates the direction of the migration of hydatid scolices from pancreatic hydatid cyst into the main pancreatic duct.

A 38-year-old man was admitted to our clinic with complaints of diffuse abdominal pain, nausea, vomiting for 7 days. The patient did not have any fever or jaundice. Moreover, he did not have any significant medical antecedents. On physical examination, vital signs were normal. Tenderness in the epigastrium was detected while examination of other systems was normal. Laboratory analyses were as follows: white blood cells were 13 000/mmc; hemoglobin was 14 g/dl; platelets were 142 000/mmc; amylase was 2100 U/l (normal value < 105); alanine aminotransferase (ALT) was 300 U/l (normal value < 40); aspartate transaminase (AST) was 120 U/l (normal value < 40); alkaline phosphatase (ALP) was 270 U/l (normal value < 290); gamma-glutamyl transpeptidase (GGT) was 130 U/l (normal value < 49); total bilirubin was 9 mg/l (normal value < 10); direct bilirubin was 3 mg/l (normal value < 8 mg/l); C-reactive protein was 20 mg/l (normal value < 5); and erythrocyte sedimentation rate was 70 mm/h. Serological tests including HBsAg, anti-HBc IgM and anti-HCV were negative. Hydatid serology, which was based on an enzyme-linked immunosorbent assay (ELISA) test for echinococcal antigens, was positive (with a value of 3,2 U/l). Lung radiography and hepatic ultrasound were normal. Abdominal computed tomography (CT) revealed a multi-loculated 100 × 90 mm cystic lesion in both the corpus and the tail of the pancreas, which was also associated with an enlargement of the pancreas and with a peripancreatic edema, indicating an acute pancreatitis. Abdominal CT-scan showed also daughter cysts, some peripheral calcifications and a detachment of the hydatid membrane in the pancreatic cyst. This is evidenced by a pressure drop inside the cyst and thus, an opening of the cyst in the pancreatic duct which is dilated (Figure 1). Nothing was detected in the liver or in any other organs. Three weeks later, the patient underwent surgery for primary pancreatic hydatid disease. Intraoperatively, following the dissection of the pancreatic tail including the cyst, a distal pancreatectomy with splenectomy was performed (Figure 2). The main pancreatic duct was disobstructed from the scolices. Histopathological examination revealed a hydatid cyst in the corpus of the pancreas opening in the main pancreatic duct (Figure 3). The region was drained and the abdomen closed. Postoperative evolution was without complication. The patient was discharged on day 6 post-operative. A 800 mg/day Albendazole therapy lasting 3 months after surgery was started on the patient. After an eight months follow-up, the patient is currently well with neither diabetes nor any signs of recurrence.

## Discussion

Pancreatic location of hydatid disease is rare (less than 1%) compared to the other sites of hydatid disease [1,2]. The mode of infestation is either hematogenous, when there is a failure of trapping oncospherse by the liver and lung filters, or more rarely through lymphatic spread [1]. The location is solitary in the pancreas in 90% of cases. The cyst can be found in the head in 50-57%, in the body in 24-34% or in the tail in 16-19% [3]. Clinical presentation varies according to the anatomic location and potential complications of the cyst (e.g. infection, rupture, biliary or intestinal fistula, segmental portal hypertension, vascular thrombosis, acute or chronic pancreatitis) [3]. With respect to the pathogenesis of pancreatitis, such as liver cysts [12,13], pancreatic hydatid cysts may cause acute pancreatitis [4-11]. While parasite migration into the common bile duct is advocated as the etiological mechanism to explain acute pancreatitis caused by liver hydatidosis, it remains unclear why some patients affected by pancreatic cysts develop this complication. Accordingly, two hypotheses are posited: main pancreatic duct compression caused by the cyst itself [7] and main pancreatic duct obstruction by hydatid scolices' migration from the hydatid cyst [6,8,9]. To date, and to the best of our knowledge, only 8 cases of acute pancreatitis due to pancreatic hydatid cyst have been reported [4-11].

The mean age of the patients was 28 years, with a range of 18-38 years. The ratio of men to women was 3. The cyst was found in the body (n = 4), tail (n = 2) or head (n = 2). The location was solitarily in the pancreas (n = 7), and associated with a liver hydatid cyst (n = 1) [9]. No specific complaints or signs at physical examination are known to distinguish hydatid cyst from other etiology of acute pancreatitis. Therefore, the final diagnosis was made only after either ultrasonography or computed tomography.

Ultrasonography will typically demonstrate a multivesicular cyst, limited by a clean wall, containing daughter cysts and some peripheral calcifications [2]. Computed tomographic findings, such as rounded cystic lesions with curvilinear calcification may allow to make the diagnosis in the appropriate clinical setting [14]. Computed tomography will also identify the prognostic stage of acute pancreatitis, which allows first, to establish the monitoring protocol, and second, to specify the time of surgery. Moreover, the abdominal CT scan can also provide indirect evidence indicating the opening of the cyst in the main pancreatic duct: the dilation of Wirsung's canal and the detachment of the hydatid membrane, which was the case in our patient. Regarding the direct sign, only Diop et al. had reported direct visualization of the migration of hydatid material from a hydatid cyst of the pancreas into the main pancreatic duct, based on data from magnetic resonance imaging and endoscopic ultrasound [9]. The cyst diameter ranged from 30 to 100 mm. In our patient, the mass size was 100 mm (missing value = 1).

Surgical treatment of hydatid pancreatic cysts may be challenging. Furthermore, depending on the cyst's location, several procedures have been suggested, ranging from cyst fenestration, internal derivation, to central or distal pancreatectomy [5-7,15-17]. As the presence of a cystopancreatic fistula may cause a long-lasting pancreatic leak after fenestration [5,16], a derivative/resective procedure is preferred in such cases. When conservative treatment is performed within local conditions that do not allow an internal derivation (inflammation seen in connection with acute pancreatitis), a possible postoperative pancreatic fistula can be treated using endoscopic retrogradecholangiopancreatography (ERCP) and placing a pancreatic stent [10]. Bedioui et al. [16] suggested intraoperative cholangiopancreatography to identify a fistula between the cyst and the main pancreatic duct, leading thus to the most appropriate surgical treatment. This diagnosis could be given preoperatively through magnetic resonance imaging or endoscopic ultrasound, allowing for planning the correct surgical strategy [9,16]. In this review of literature, procedures that have been performed were as following: left pancreatectomy (n = 5) from which one was with splenic preservation, cyst fenestration (n = 2) and total cystectomy (n = 1). No recurrence was diagnosed after a mean of 13 month (missing value = 1).

## Conclusion

Hydatid cyst of the pancreas is an extremely rare pathology but it may be a causal factor in acute pancreatitis, especially in endemic areas. Radiological examinations may help clinicians in diagnosing cystic masses in the pancreas. In the case of acute pancreatitis due to pancreatic hydatid cyst, there is definitely a closed relationship between the main pancreatic duct and the pancreatic cyst, imposing a derivative or a resective procedure.

## Consent

Written informed consent was obtained from the patient for publication of this case report and accompanying images. A copy of the written consent is available for review by the Editor-in-Chief of this journal.

## Competing interests

The authors declare that they have no competing interests.

## Authors' contributions

AM prepared the manuscript and performed the literature review. MJ formulated and assisted in the preparation of the manuscript. AM and MK conceived and performed the technique described in this manuscript. ZBS had given final approval of the version to be published. All authors have read and approved the final manuscript.
